# Synthesis and Characterization of Random Block Hydroxyl-Terminated Polyfluoroether-Based Polyurethane Elastomers with Fluorine-Containing Side Chains

**DOI:** 10.3390/polym15020288

**Published:** 2023-01-06

**Authors:** Yanqiu Zhou, Junjie Chen, Limin Zhang, Hui Huang, Rufang Peng, Bo Jin

**Affiliations:** 1 State Key Laboratory of Environment-Friendly Energy Materials, School of Materials and Chemistry, Southwest University of Science and Technology, Mianyang 621010, China; 2 China Academy of Engineering Physics, Mianyang 621900, China

**Keywords:** fluoropolymer, polyurethane elastomers, mechanical property, thermal behavior

## Abstract

Polype ntafluoropropane glycidyl ether (PPFEE), a new random block hydroxyl-terminated polyfluoroether, was synthesized successfully by cationic ring-opening polymerization of 2-(2,2,3,3,3-pentafluoropropoxymethyl) oxirane, and its molecular structure was confirmed by Fourier transform infrared spectroscopy, nuclear magnetic resonance spectrometry, and gel permeation chromatography. The PPFEE-based polyurethane elastomers featuring fluorine in their side chains were prepared using PPFEE as soft segments, polyisocyanate polyaryl polymethylene isocyanate as hard segments, and dibutyltin dilaurate as catalysts under different curing conditions. The microphase separation, mechanical performance, and thermal behavior of the elastomers were investigated by differential scanning calorimetry, uniaxial tensile test, and thermal gravimetric analysis, respectively. Based on the results, the percentage of hard segments dissolved into the soft segments of elastomers was opposite to the change in breaking strength. The PPFEE-based polyurethane elastomer cured with 20 wt% PAPI at the curing temperature of 50 °C displayed the maximum tensile elongation of 2.26 MPa with an elongation at break of nearly 150%. The increased contents of PAPI can effectively strengthen the tensile strength, and the maximum tensile elongation was 3.04 MPa with an elongation at break of nearly 90% when the content of PAPI was 26 wt%. In addition, the PPFEE-based polyurethane elastomers exhibited excellent resistance to thermal decomposition and a sharp weight loss temperature at around 371 °C. All the results demonstrated that the PPFEE may be a potential polymeric binder as one of the ingredients applied to future propellant formulations.

## 1. Introduction

Polymeric binders are one of the important components of solid propellants, and they can bind numerous metallic fuels together with oxidizers, plasticizers, and other additives to form integration in solid propellants [1,2,3]. In addition, the integrated structure must be prepared into a certain shape that can withstand a certain degree of stress. In general, the structural integrity is affected by the characteristics of the polymeric binder.

Fluorine is the most electronegative element [4], and fluorinated compounds are commonly used as oxidants. Therefore, the introduction of fluorine to polymeric binders can improve the oxygen balance of solid propellants, resulting in less use of oxidants and increased utilization of energetic materials, which can enhance the energy level of solid propellants [5]. In recent decades, owing to their numerous attractive properties, including high density, low coefficient of friction, long-term chemical stability, broad operating temperature range, and good compatibility with other main ingredients [6,7,8,9], fluoropolymers have emerged as active polymeric binders, and have become one of the most widely applied solid propellants [10,11,12,13,14,15,16,17]. In addition, fluorinated polyurethane elastomers possess unique mechanical properties [18,19,20] by the virtue of the influences of fluorine on the microstructure with separate hard and soft phases. In general, the fluorine in soft segments can enhance the interaction between hard and soft segments, reducing the microphase separation degree.

In this study, polypentafluoropropane glycidyl ether (PPFEE), a new random block hydroxyl-terminated polyfluoroether, was synthesized through cationic ring-opening polymerization of 2-(2,2,3,3,3-pentafluoropropoxymethyl) oxirane (PFEE) using butane-1,4-diol (BDO) as the initiator and boron trifluoride diethyl ether (BF_3_·OEt_2_) as the catalyst. The molecular structure of PPFEE was characterized by Fourier transform infrared spectroscopy (FT-IR), nuclear magnetic resonance spectrometry (NMR), and gel permeation chromatography (GPC). Then, the PPFEE-based polyurethane elastomers featuring fluorine (F) in the side chains were prepared using PPFEE as the prepolymer, polyisocyanate polyaryl polymethylene isocyanate (PAPI) as the crosslinking agent, and dibutyltin dilaurate (DBTDL) as the catalyst. The microphase separation, mechanical performance, and thermal behavior of PPFEE-based polyurethane elastomers were characterized by differential scanning calorimetry (DSC), uniaxial tensile test, and thermal gravimetric analysis (TGA), respectively.

## 2. Experimental Section

### 2.1. Materials

PAPI, whose isocyanate content was 30.87 g/100 g, was purchased from Zhengzhou Keyunlong Chemical Products Co., Ltd. (Zhengzhou, China) and used without further purification. 2,2,3,3,3-Pentafluoropropanol and epichlorohydrin (ECH) were purchased from Aladdin (Shanghai, China). BF_3_·OEt_2_, phthalic anhydride, DBTDL, sodium hydroxide (NaOH), sodium sulfate (Na_2_SO_4_), and methylene chloride (CH_2_Cl_2_) were purchased from Chengdu, Kelong Chemical Reagent Factory (Chengdu, China). BDO was purchased from Tokyo Chemical Industry Co., Ltd. (Shanghai, China). All the reactants were used without further purification.

### 2.2. Synthesis of PFEE

**Scheme 1:**
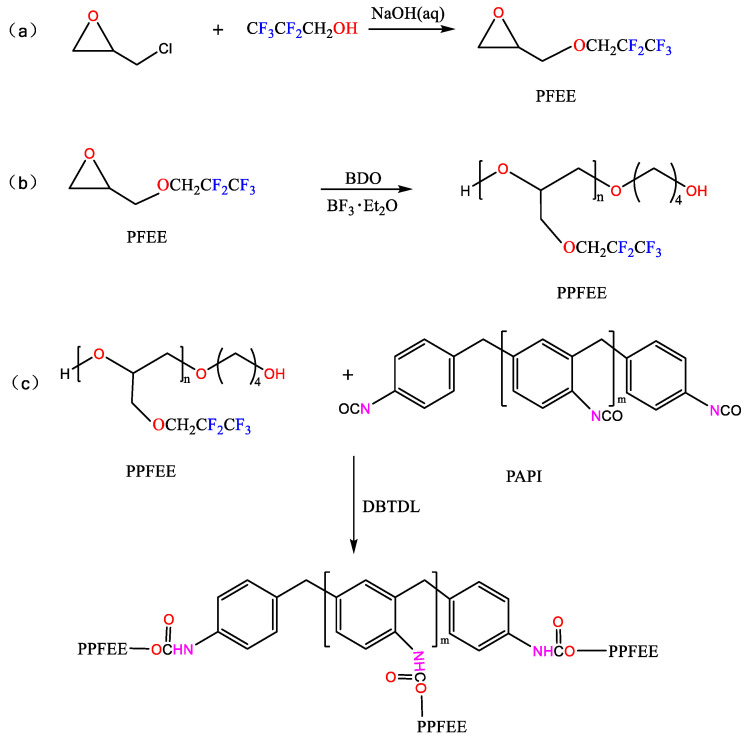
Synthesis routes of PFEE (**a**), PPFEE prepolymer (**b**), and PPFEE-based polyurethane elastomers (**c**).

PFEE was first synthesized from ECH and 2,2,3,3,3-pentafluoropropanol, referring to a procedure in previous literature [21] (Scheme 1a). 2,2,3,3,3-Pentafluoropropanol (30 g, 0.2 mol), ECH (92 g, 1.0 mol), sodium hydroxide (12 g, 0.3 mol), and a catalytic amount of water (0.35 mL) were successively weighed into a dry 250 mL three-necked round-bottom flask equipped with a magnetic stirrer, condenser, and thermometer. After constantly stirring for an additional 7 h at 70 °C, the reaction mixture was evaporated under reduced pressure. Next, the pure PFEE was obtained as the collected fraction through distillation at 108 °C–112 °C. Its structure was confirmed through ^1^H NMR and ^13^C NMR.

^1^H NMR (600 MHz, CDCl_3_, δ): 4.09–3.89 (m, 2H, CH_2_ in O-CH_2_-CF_2_ and one molecule of CH_2_ in CH-CH_2_-O), 3.56–3.47 (m, 1H, one molecule of CH_2_ in CH-CH_2_-O), 3.21–3.15 (m, 1H, CH), 2.82–2.77 (m, 1H, one molecule of CH_2_ in ring), and 2.63–2.60 (m, 1H, one molecule of CH_2_ in ring); ^13^C NMR (151 MHz, CDCl_3_, δ): 121.72–115.58 (CF_2_), 115.01–110.89 (CF_3_), 72.94 (CH_2_ in CH-CH_2_-O), 67.82–67.47 (CH_2_ in O-CH_2_-CF_2_), 50.32 (CH), and 43.43(CH_2_ in the ring).

### 2.3. Synthesis of PPFEE Prepolymer

PPFEE was obtained via the typical cationic ring-opening polymerization of PFEE as described in Scheme 1b. The reaction steps involved are described below. BDO (256 μL) and BF_3_·OEt_2_ (175 μL) dissolved in methylene chloride (12 mL) were contained in a flask and stirred at room temperature for 0.5 h. After cooling the reaction vessel to 0 °C and replacing the atmosphere with nitrogen, PFEE (30 g) was slowly and dropwise added to the reaction mixture over a period of 12 h. When the addition was completed, the reaction was continued under stirring at room temperature for an additional 12 h. After polymerization, the reaction was terminated through the addition of distilled water. The organic phase containing PFEE was extracted into methylene chloride and washed with distilled water several times until neutral pH was obtained. The washed organic phase was dried over sodium sulfate and filtered. Finally, the solvent was evaporated off in a 120 °C vacuum to afford 25 g PPFEE (83% yield). The hydroxyl value was 0.54 mmol g^−1^. The weight-average molecular weight (*M_w_*), the number-average molecular weight (*M_n_*), and the polydispersity index (PDI) of PFEE from GPC were 3911 g mol^−1^, 3307 g mol^−1^, and 1.18, respectively.

### 2.4. Preparation of PPFEE-Based Polyurethane Elastomers

PPFEE prepolymer was dehydrated in a vacuum oven at 80 °C for 48 h before use. PPFEE-based polyurethane elastomers with various mass percentages of PAPI or at different curing temperatures were prepared using the dried PPFEE prepolymer as soft segments, PAPI as hard segments, and DBTDL as catalysts. The detailed preparation procedure was as follows. The same masses of PPFEE and DBTDL (0.4 wt%) were mixed in four 50 mL beakers. After stirring for 10 min, different contents of PAPI (17, 20, 22, and 26 wt%) were added respectively, and the mixtures were stirred for another 10 min until a uniformly yellow fluid was formed, followed by degassing in a vacuum oven. Afterward, the mixtures were transferred to four round-shaped Teflon-coated molds followed by a reaction for 2 days at 20 °C and then curing for 2 days at 50 °C. Meanwhile, the mixtures of PPFEE and DBTDL with the same mass as before and 20 wt% PAPI were left to react for 2 days at 20 °C and then cured for 2 days at 40 °C, 50 °C, 60 °C, and 70 °C. The dumbbell-shaped specimens of PPFEE-based polyurethane elastomers were prepared to measure the mechanical properties, glass transition temperature, and thermal stability through the universal testing machine, DSC, and TGA, respectively.

### 2.5. Characterization

FTIR spectra were recorded in a Nicolet 380 FTIR spectrophotometer (Thermo Fisher Nicolet, Waltham, MA, USA) with a wavenumber resolution of 4 cm^−1^ in the range from 450 cm^−1^ to 4000 cm^−1^. ^1^H NMR and ^13^C NMR were measured with AVANCE III 600 MHz Bruker instrument (Switzerland) using CDCl_3_ as the solvent. ^19^F NMR was conducted on a 400 MHz Bruker spectrometer using CDCl_3_ as solvent. GPC was conducted on a Waters GPC 1515 instrument (USA).

DSC experiments conducted with DSC Q200 produced by TA Instruments (USA) were used to thermally characterize the samples, which utilized heating ramps of 10 °C min^−1^ under a dry nitrogen atmosphere (50 mL/min). A second scan from −70 to 20 °C was recorded. Mechanical properties, including tensile strength and elongation at break, of all the polyurethane elastomers gels, were measured on an HS-20KN universal testing machine produced by Yangzhou Huahui Testing Instrument Co., Ltd. (Yangzhou, China) with a tensile rate of 500 mm min^−1^. TGA performed on Netzsch STA 409 PC simultaneous thermal analysis instrument (Germany) was used to characterize the thermal decomposition properties of the polyurethane elastomers gels in the temperature range from 50 °C to 800 °C with the heating rate of 10 °C min^−1^.

## 3. Results and Discussion

### 3.1. Structure of PPFEE

**Figure 1:**
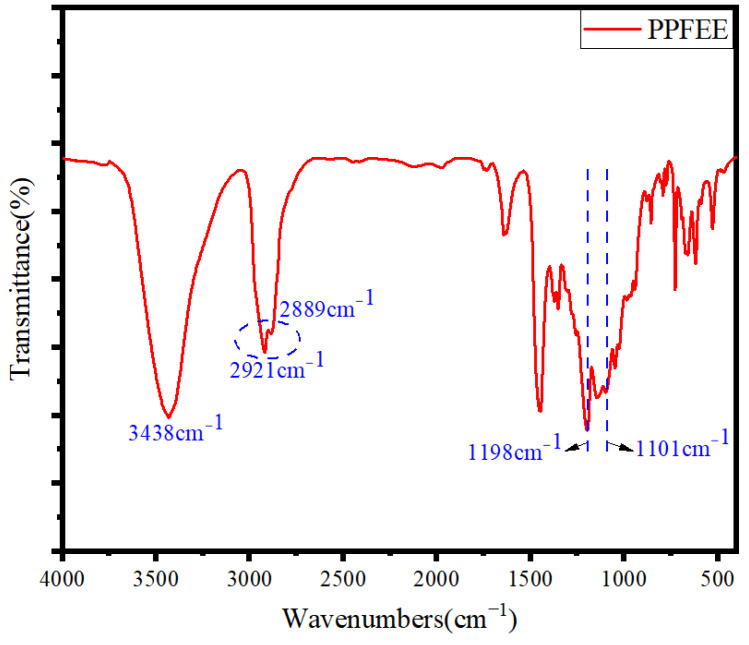
FTIR spectra of PPFEE.

The PPFEE-based polyurethane elastomers were prepared via a prepolymer process (Scheme 1). The FTIR spectrum provided the first confirmation of the PPFEE structure. As shown in Figure 1, the strongest peak at 3438 cm^−1^ corresponded to O-H stretching vibration, and the bands around 2921 and 2889 cm^−1^ were related to -CH_2_- stretching vibrations. The stronger peak at 1198 cm^−1^ was ascribed to C-O-C, and the bands attributed to C-F stretching vibrations were observed at 1101 cm^−1^.

**Figure 2:**
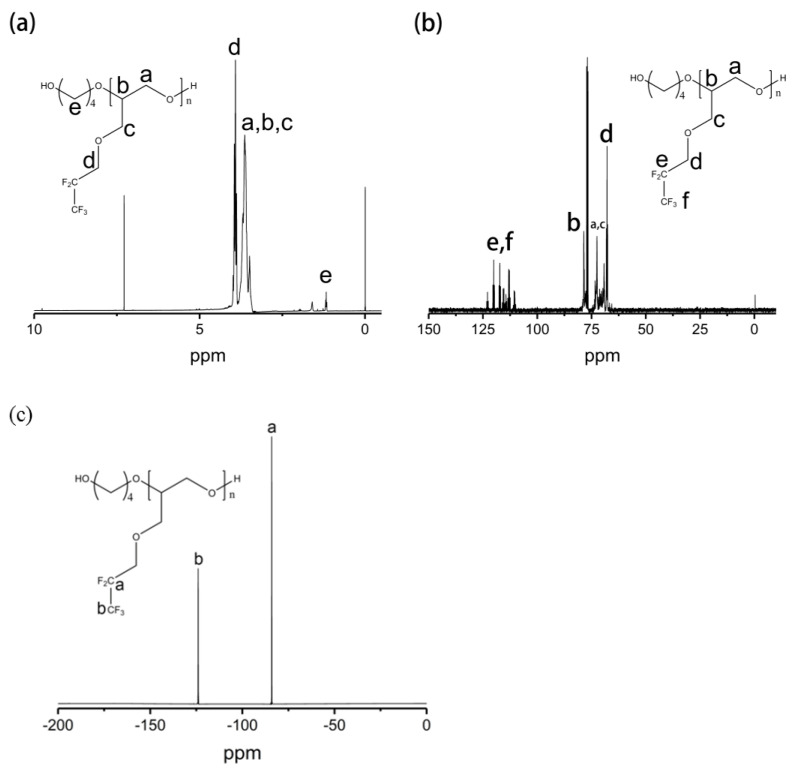
^1^H NMR (**a**), ^13^C NMR (**b**), and ^19^F NMR (**c**) spectra of PPFEE in CDCl_3_.

The PPFEE structure was further confirmed using NMR analysis. The ^1^H NMR spectrum of PPFEE in Figure 2a shows the triple peak observed at δ: 3.95, 3.92, and 3.88 ppm, which were attributed to the methylene protons (d) of the side chains, signals at 3.81–3.45 ppm related to methylene protons of the main (a and b) and side (c) chains, and the multiple peaks at 1.19–1.16 ppm ascribed to the initiator in the main chain, respectively. According to the ^13^C NMR of PPFEE shown in Figure 2b, the multiple peaks observed at 123.28–114.07 and 116.11–109.94 ppm were ascribed to the carbons in -CF_3_ (f) and in -CF_2_ (e), respectively. The signal observed at 78.61–78.38 ppm was attributed to CH in the main chain (b). The signal detected at 73.61–69.27 ppm was related to CH_2_ in the main (a) and side (c) chains. The triple peaks identified at 68.14, 67.68, and 67.62 ppm were assigned to -CH_2_- in the side chain (d). Figure 2c shows the ^19^F NMR spectrum of PPFEE. The signals observed at −84.80 and −124.00 ppm were due to -CF_2_ (a) and -CF_3_ (b), respectively. Both signal positions observed in the FTIR and NMR spectra confirmed the successful synthesis of PPFEE.

### 3.2. Glass Transition Temperature (T_g_) Measurement

**Figure 3:**
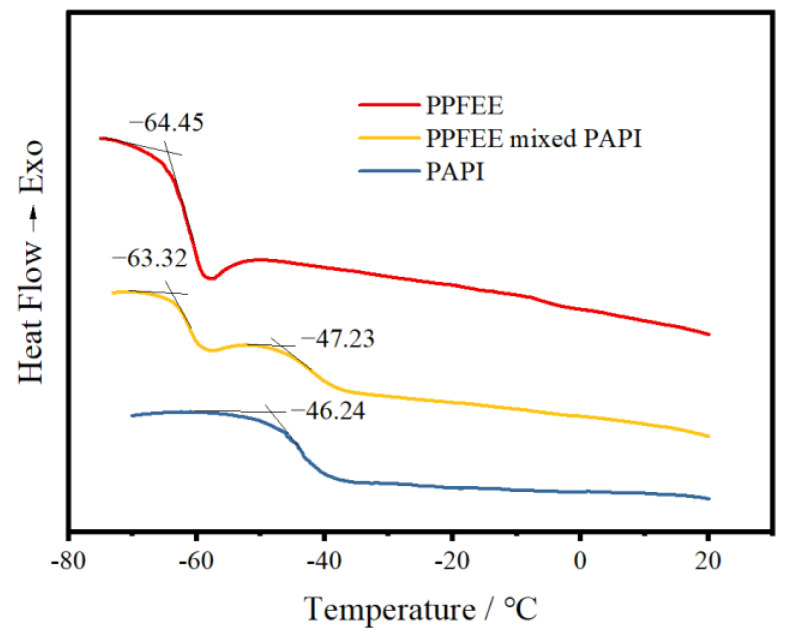
DSC curves of PAPI, PPFEE, and PPFEE/PAPI mixture.

The *T_g_* is one of the most important parameters of polymeric binders because it has a decisive influence on mechanical properties at low temperatures [22]. Figure 3 shows the second-heating DSC curves of the PAPI, PPFEE, and PPFEE/PAPI mixture. The PPFEE exhibited a low *T_g_* of −64.45 °C, which was beneficial for low-temperature properties. The PPFEE/PAPI mixture showed two *T_g_* indicating their incompatibility.

### 3.3. Mechanical Properties of Polyurethane Elastomers

**Scheme 2:**
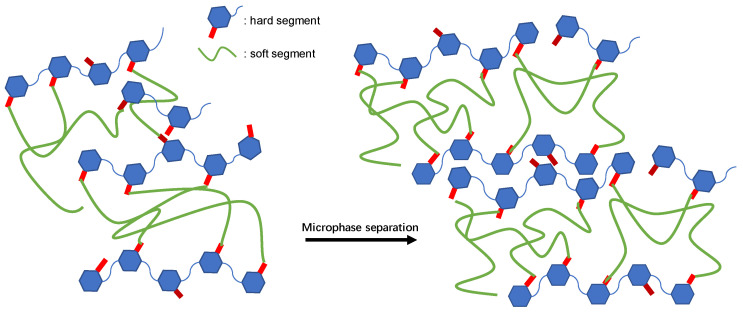
Schematic of microphase separation.

**Figure 4:**
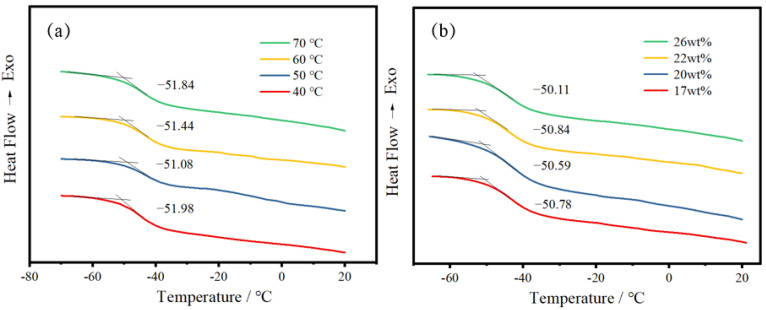
*T_g_* of PPFEE-based polyurethane elastomers cured with 20 wt% PAPI at different temperatures (**a**) and with different amounts of PAPI content at 50 °C (**b**).

The microphase-separated microstructures in the PPFEE-based elastomers were expected to affect their mechanical properties. In essence, the microphase separation is a phase separation equilibrium process (Scheme 2) [20]. According to the microphase separation theory, a certain number of hard and soft segments can be dissolved into each other, and these mutual solubility results can change the *T_g_* of the two phases based on the mode of the copolymer equation. Thus, the *T_g_* of the soft link phase will increase, and that of hard segments will decrease. In general, the proportion of soft segments dissolved into the microdomain of hard segments is relatively small, and only the case where hard segments are dissolved into the soft segments is considered. Therefore, by studying the *T_g_* of soft segments, the phase separation degree of elastomers can be characterized. This change can be described by the Gordon–Taylor equation (Formula (1)) [23].

(1) $$\frac{1}{T_{g}}=\frac{W_{1}}{T_{g1}}+\frac{W_{2}}{T_{g2}}$$

where *T_g_*_1_ and *T_g_*_2_ represent the *T_g_* of pure soft and hard segments, respectively. *W*_1_ and *W*_2_ denote the percentage of the soft segments and hard segments dissolved into the soft segment phase in the soft segment phase, respectively, *W*_1_ + *W*_2_ = 1. *T_g_* was measured through DSC (Figure 4), and it was considered the value of the soft segment phase giving an estimate of the extent of hard segments dissolved in soft segment domains.

Glass transitions are associated with the movement of polymer chain segments due to intermolecular interactions. The interaction between hard and soft segments can be indicated through the analysis of changes in the *T_g_* of samples prepared under different conditions. Figure 4a shows that *T_g_* increased first and then decreased with the increase in the curing temperature. The *T_g_* at the curing temperature of 50 °C was the maximum owing to the central arrangement of hard segments, which was consistent with the results for the tensile strength. As shown in Figure 4b, the *T_g_* of PPFEE-based polyurethane elastomers increased slightly with the increased contents of hard segments due to the increase in the percentage of hard segments dissolved in the soft segment domains.

In addition, -NH- in the hard segments can create a hydrogen bond with O in the soft segments, which may greatly limit the movement of soft segments and thus increase their *T_g_* [24,25,26]. Therefore, considering the influence of hard segments on the *T_g_* of soft segments after the dissolution of hard segments into the soft segment phase, the effect of physical crosslinking was also accounted for. The effects can be described by the Dibenedetto and Dimarzio equations (Formula (2)).

(2) $$\frac{T_{g}-T_{g1}}{T_{g1}}=\frac{kX_{c}}{1-X_{c}}$$

where constant *k* is 0.1; *X_c_* is the mole fraction of soft segment structural units forming hydrogen bonds.

(3) $$Xc=\frac{W_{2}M_{1}}{W_{1}M_{2}}$$

where *M*_1_ and *M*_2_ are the relative molar masses of soft and hard segment structural units, respectively.

We obtained the following after the transformation of Formula (1):

(4) $$\frac{T_{g}-T_{g1}}{T_{g1}}=\frac{T_{g2}}{T_{g2}-(T_{g2}-T_{g1})W_{2}}-1$$

From Formula (3), we arrived at the equation below:

(5) $$W_{2}=\frac{M_{2}X_{c}}{M_{1}+M_{2}X_{c}}$$

Incorporating Formula (5) into Formula (4), we obtained the following:

(6) $$\frac{T_{g}-T_{g1}}{T_{g1}}=\frac{M_{2}X_{c}(T_{g2}-T_{g1})}{T_{g2}(M_{1}+M_{2}X_{c})-(T_{g2}-T_{g1})M_{2}X_{c}}$$

Assuming that the effects of copolymerization and crosslinking on the *T_g_* can be added linearly, we combined Equations (2) and (6) [27]:

(7) $$\frac{T_{g}-T_{g1}}{T_{g1}}=\frac{M_{2}X_{c}(T_{g2}-T_{g1})}{T_{g2}(M_{1}+M_{2}X_{c})-(T_{g2}-T_{g1})M_{2}X_{c}}+\frac{kX_{c}}{1-X_{c}}$$

Given that polyether polyurethane elastomer has a good degree of microphase separation, the ratio of the hard segment phase dissolved into the soft segment phase (*W*_2_) is generally small, and the corresponding *X_c_* is also small. To simplify the calculation, ignoring the high item (Formula (7)), we obtained the equation (Formula (8)) that affects the *T_g_* of the soft segment phase as follows, and *X_c_* was obtained through the equation:

(8) $$\frac{T_{g}-T_{g1}}{T_{g1}}=\frac{(kT_{g2}M_{1}+M_{2}T_{g2}-M_{2}T_{g1})X_{c}}{T_{g2}M_{1}+(T_{g1}M_{2}-T_{g2}M_{1})X_{c}}$$

Let *W* be the percentage of hard segments dissolved into the soft segments in the elastomers, and *H* is the percentage of hard segments in the elastomers. Then, we obtained the following:

(9) $$W_{2}=\frac{HW}{(1-H)+HW}$$

Substituting Formula (9) into Formula (5) and after mathematical transformation, the following equation was solved:

(10) $$W=\frac{(1-H)M_{2}X_{c}}{M_{1}H}$$

The calculated results are listed in Table 1 and Table 2. The percentage of hard segments dissolved into soft segments (W) was proportional to the *T_g_*, and the minimum values were observed at 50 °C at 26% PAPI, indicating that the extent of microphase separation reached the maximum which had a great influence on the mechanical properties.

**Figure 5:**
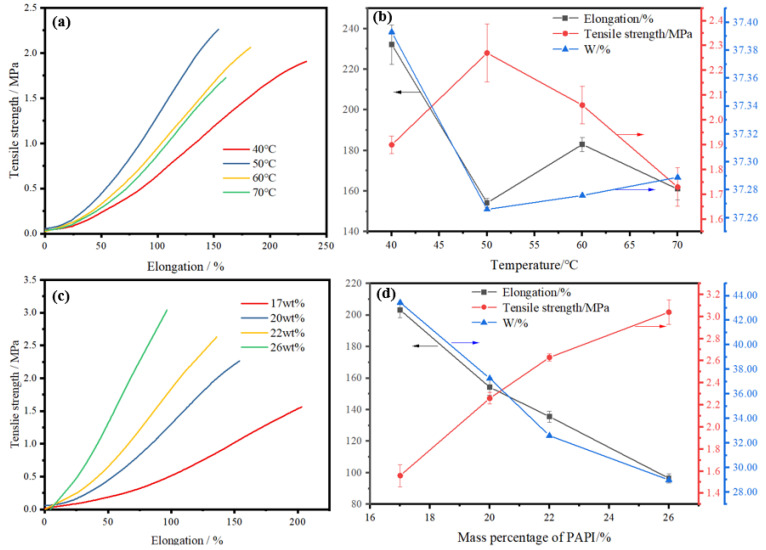
Mechanical properties of PPFEE-based polyurethane elastomers cured with 20 wt% PAPI at different temperatures (**a**,**b**) and with different amounts of PAPI content at 50 °C (**c**,**d**) (five splines for each curing condition were used as parallel tests, and the most reproducible data as the final result).

The nature of binder systems plays a decisive role in the mechanical properties of composite propellants [28]. Figure 5a depicts the stress-strain curves of PPFEE cured with 20 wt% PAPI at different temperatures. The PPFEE-based polyurethane elastomers gave the maximum tensile strength of 2.26 MPa with an elongation at break of nearly 150% at the curing temperature of 50 °C, and this finding was attributed to the possible reason for the poor compatibility between soft and hard segments, resulting in the maximum extent of microphase separation. Thus, the mechanical properties of cured splines were greatly affected by the degree of microphase separation. However, the hard segment content may play a critical role when the curing temperature is constant. The stress-strain curves (Figure 5c) for four test series of PPFEE cured with various contents of PAPI at 50 °C were depicted. As anticipated, the tensile strength increased, and elongation at break correspondingly decreased along with the increase in the hard segment content. The maximum tensile strength was 3.04 MPa, that is, an elongation at break of nearly 90%. Moreover, as shown in Figure 5b,d, the most reproducible data with a standard deviation of tensile strength and elongation at break were provided. The tensile strength with the largest dispersion degree reached the maximum at 50 °C (seen in Figure 5b), which was still in the acceptable range. The result indicated that with the increase in temperature, the tensile strength increased and then decreased. The tensile strength reached the maximum when the content of PAPI was 26 wt% (seen in Figure 5d). This result indicated that with the increase in the content of PAPI, the tensile strength increased in direct proportion. In addition to these, the variation in the tensile strength of the splines was opposite the trend of W representing the ratio of hard segments dissolved into the soft segment phase. The reason may be that the elastomers, whose hard segments were dispersed into the soft segment matrix, had no obvious hindrance to the deformation under external stress [29].

### 3.4. Thermal Decomposition

**Figure 6:**
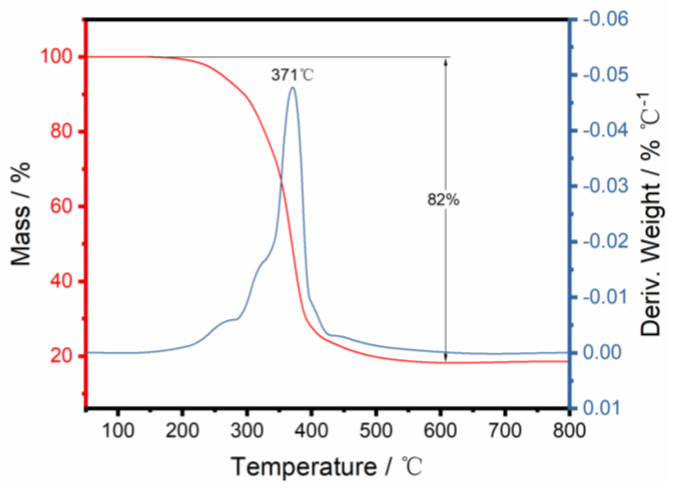
Thermogravimetry/derivative thermogravimetry (TG/DTG) curves of the PPFEE-based polyurethane elastomer cured with 26 wt% PAPI at 50 °C.

The thermal stability of polymeric binders is a critical property for their application in solid propellants [30,31,32]. Thus, TG and DTG were used to study the thermal decomposition behavior of polyurethane elastomers (Figure 6). The TG curve showed one distinct region of weight loss in the thermal decomposition of the PPFEE-based polyurethane elastomer. The stage presented a slow weight loss starting at nearly 200 °C, and this result corresponded to the decomposition of the end groups and a small number of side chains. Then, the main chain decomposed, and the weight loss rate gradually increased. The weight loss temperature was observed at 371 °C with a sharp weight loss. After the complete thermal decomposition, the remaining residue was approximately 18%. In any case, the TG/DTG results confirmed that PPFEE-based polyurethane elastomers had satisfactory thermal stability.

## 4. Conclusions

A novel random block hydroxyl-terminated polyfluoroether binder, PPFEE, was synthesized through typical cationic ring-opening polymerization. From the FTIR, NMR, and GPC results, PPFEE was synthesized successfully via the synthesis route. The DSC curves indicated that PPFEE had a low *T_g_* of −64.45 °C. Through DSC research, the percentage of hard segments dissolved into the soft segments of elastomers exerted important influences on the mechanical properties. The PPFEE-based polyurethane elastomer cured with 20 wt% PAPI at 50 °C displayed the maximum tensile strength of 2.26 MPa with elongation at break of nearly 150%. The increased contents of PAPI can effectively enhance the tensile strength, and the maximum tensile strength was 3.04 MPa with an elongation at break of nearly 90% when the content of PAPI was 26 wt%. The TG/DTG curves displayed adequate resistance to thermal decomposition up to 200 °C, and the weight loss temperature was observed at 371 °C with a sharp weight loss. All of these results indicated that PPFEE may be a potential polymeric binder as one of the ingredients applied to future propellant formulations.

## Figures and Tables

**Table 1:** Values of *T_g_* and *W* at different temperatures.

*T*/°C	40	50	60	70
***T_g_*/°C**	−51.98	−51.08	−51.44	−51.84
***W*/%**	37.293	37.266	37.276	37.289

**Table 2:** Values of *T_g_* and *W* at various mass percentages of PAPI.

**Mass Percentage/%**	17	20	22	26
***T_g_/*°C**	−50.11	−50.84	−50.59	−50.78
***W*/%**	43.447	37.266	32.596	28.989

## Data Availability

The data presented in this study are available on request from the corresponding author.

## References

[1] Kubota N., Kuwahara T., Miyazaki S., Uchiyama K., Hirata N. (1986). Combustion wave structures of ammonium perchlorate composite propellants. J. Propuls. Power.

[2] Liu F.B., Zhang X.L., Jiang W.S., Yu F.Y., Deng J.R. (2018). Study on the curing system of polytriazole adhesive for composite solid propellant. Propellants Explos. Pyrotech..

[3] Guery J.F., Chang I.S., Shimada T., Glick M., Boury D., Robert E., Napior J., Wardle R., Pérut C., Calabro M. (2010). Solid propulsion for space applications: An updated roadmap. Acta Astronaut..

[4] Jaccaud M., Faron R., Devilliers D., Romano R. (2000). Fluorine. Ullmann’s Encyclopedia of Industrial Chemistry.

[5] Cheng T. (2019). Review of novel energetic polymers and binders–high energy propellant ingredients for the new space race. Des. Monomers Polym..

[6] Lee I., Reed R.R., Brady V.L., Finnegan S.A. (1997). Energy release in the reaction of metal powders with fluorine containing polymers. J. Therm. Anal. Calorim..

[7] Lee J.H., Kim S.J., Park J.S., Kim J.H. (2016). Energetic Al/Fe_2_O_3_/PVDF composites for high energy release: Importance of polymer binder and interface. Macromol. Res..

[8] Dattelbaum D.M., Sheffield S.A., Stahl D., Weinberg M., Neel C., Thadhani N. (2008). Equation of state and high pressure properties of a fluorinated terpolymer: THV 500. J. Appl. Phys..

[9] Rider K.B., Little B.K., Emery S.B., Lindsay C.M. (2013). Thermal analysis of magnesium/perfluoropolyether pyrolants. Propellants Explos. Pyrotech..

[10] Zhang T., Zhang W., Liu H., Wang G., Zhong Y., Zhou M., Zhu Q., Li H. (2021). Synthesis and characterization of a novel fluorine-containing triblock copolymer as a potential binder. Eur. Polym. J..

[11] Xu M., Ge Z., Lu X., Mo H., Ji Y., Hu H. (2017). Structure and mechanical properties of fluorine-containing glycidyl azide polymer-based energetic binders. Polym. Int..

[12] Xu M., Ge Z., Lu X., Mo H., Ji Y., Hu H. (2017). Fluorinated glycidyl azide polymers as potential energetic binders. RSC Adv..

[13] Zhang T., Liu H., Shuai J., Gao Z., Dong Q., Wang G., Xiong Y., Zhu Q., Li H. (2021). Synthesis and characterization of a novel fluorine-containing copolymer P(FPO/NIMMO) as a potential energetic binder. J. Fluor. Chem..

[14] Xu M., Lu X., Liu N., Zhang Q., Mo H., Ge Z. (2021). Fluoropolymer/Glycidyl Azide Polymer (GAP) Block Copolyurethane as New Energetic Binders: Synthesis, Mechanical Properties, and Thermal Performance. Polymers.

[15] Zhang W., Zhang T., Liu H., Zheng Y., Zhong Y., Wang G., Zhu Q., Liu X., Zhang L., Li H.l. (2022). Synthesis and characterization of a novel hydroxy telechelic polyfluoroether to enhance the properties of HTPB solid propellant binders. Colloids Surf. A Physicochem. Eng. Asp..

[16] Lan Q., Kim J.S., Kwon Y. (2016). Synthesis and Thermal Characteristics of Nano-Aluminum/Fluorinated Polyurethane Binders. J. Korean Soc. Propuls. Eng..

[17] Zhang X., Kim J.S., Kwon Y. (2017). Synthesis and thermal analysis of nano-aluminum/fluorinated polyurethane elastomeric composites for structural energetics. J. Nanosci. Nanotechnol..

[18] Wang X., Hu J., Li Y., Zhang J., Ding Y. (2015). The surface properties and corrosion resistance of fluorinated polyurethane coatings. J. Fluor. Chem..

[19] Yu Y., Chen S., Li X., Zhu J., Liang H., Zhang X., Shu Q. (2016). Molecular dynamics simulations for 5,5′-bistetrazole-1,1′-diolate (TKX-50) and its PBXs. RSC Adv..

[20] Wang X., Xu J., Li L., Liu Y., Li Y., Dong Q. (2016). Influences of fluorine on microphase separation in fluorinated polyurethanes. Polymer.

[21] Matuszczak S., Feast W.J. (2000). An approach to fluorinated surface coatings via photoinitiated cationic cross-linking of mixed epoxy and fluoroepoxy systems. J. Fluor. Chem..

[22] Wang F.X., Jin B., Peng R., Zhang Q., Gong W. (2015). Synthesis, spectroscopic characterization, thermal stability and compatibility properties of energetic PVB-gGAP copolymers. J. Polym. Res..

[23] Gordon J.M., Rouse G.B., Gibbs J.H., Risen W.M. (1977). The composition dependence of glass transition properties. J. Chem. Phys..

[24] Ampleman G., Beaupre F. Synthesis of linear GAP based energetic thermoplastic elastomers for use in HELOVA gun propellant formulations. Proceedings of the 27th International Annual Conference of ICT.

[25] Mulage K.S., Patkar R.N., Deuskar V.D., Pundlik S.M., Kakade S.D., Gupta M. (2007). Studies on a novel thermoplastic polyurethane as a binder for extruded composite propellants. J. Energetic Mater..

[26] Mirhosseini M.M., Haddadi-Asl V., Jouibari I.S. (2019). How the soft segment arrangement influences the microphase separation kinetics and mechanical properties of polyurethane block polymers. Mater. Res. Express.

[27] Chen C.H., Briber R.M., Thomas E.L., Xu M., MacKnight W.J. (1983). Structure and morphology of segmented polyurethanes: 2. Influence of reactant incompatibility. Polymer.

[28] Wang L., Song Y., Gyanda R., Sakhuja R., Meher N.K., Hanci S., Gyanda K., Mathai S., Sabri F., Ciaramitaro D.A. (2010). Preparation and mechanical properties of crosslinked 1, 2, 3-triazole-polymers as potential propellant binders. J. Appl. Polym. Sci..

[29] Hu Y., Jian X., Xiao L., Zhou W. (2018). Microphase separation and mechanical performance of thermoplastic elastomers based on poly (glycidyl azide)/poly (oxytetramethylene glycol). Polym. Eng. Sci..

[30] Hanafi S., Trache D., He W., Xie W.X., Mezroua A., Yan Q.L. (2020). Thermostable energetic coordination polymers based on functionalized go and their catalytic effects on the decomposition of ap and rdx. J. Phys. Chem. C.

[31] Tarchoun A.F., Trache D., Klapötke T.M., Belmerabet M., Abdelaziz A., Derradji M., Belgacemi R. (2020). Synthesis, characterization, and thermal decomposition kinetics of nitrogen-rich energetic biopolymers from aminated giant reed cellulosic fibers. Ind. Eng. Chem. Res..

[32] Hanafi S., Trache D., Meziani R., Boukciat H., Mezroua A., Tarchoun A.F., Derradji M. (2021). Synthesis, characterization and thermal decomposition behavior of a novel HNTO/AN co-crystal as a promising rocket propellant oxidizer. Chem. Eng. J..

